# An update on the combined surgical and orthodontic treatment of complex odontoma among growing patients young patients

**DOI:** 10.6026/973206300211729

**Published:** 2025-05-31

**Authors:** Shruti Garg, Kanika Arora, Komil Tintodana, John Kujur, Ipsita Roy, Neha Agrawal

**Affiliations:** 1 Department of Orthodontics and Dentofacial Orthopedics, Modern Dental College and Research Centre, Indore, Madhya Pradesh, India; 2 Department of Orthodontics and Dentofacial Orthopedics, Manav Rachna Dental College, Faridabad, Haryana, India; 3 Department of Orthodontics and Dentofacial Orthopedics, Siddhpur Dental College and Hospital, Gujarat, India; 4 Department of Prosthodontics and Crown and Bridge, Hi Tech Dental College, Bhubaneswar, Odisha, India; 5 Department of Orthodontics and Dentofacial Orthopedics, Kalinga Institute of Dental Sciences, KIIT University, Odisha - 751024, India; 6 Department of Biochemistry, Index Medical College Hospital and Research Center, Indore, Madhya Pradesh, India

**Keywords:** Complex odontoma, odontogenic tumor, surgical enucleation, orthodontic traction, impacted teeth, pediatric dentistry, interdisciplinary dental treatment, tooth eruption, dental anomalies, growing patients, mixed dentition, maxillofacial surgery, orthodontic management, dental radiology, odontoma eruption guidance

## Abstract

Complex odontomas are benign odontogenic hamartomas that frequently hinder the eruption of permanent teeth in growing individuals. Therefore, it is of interest to evaluate the clinical effectiveness of a coordinated surgical and orthodontic treatment protocol in 20 patients aged 8-16 years diagnosed with complex odontomas. Surgical excision was followed by orthodontic intervention to facilitate eruption and alignment of impacted teeth. Successful eruption was observed in 90% of cases within 6-12 months, with favourable alignment outcomes and no recurrence over the 12-month follow-up. These findings affirm the value of early diagnosis and interdisciplinary management in optimizing functional and esthetic outcomes in pediatric patients with complex odontomas.

## Background:

Complex odontomas are developmental malformations-classified as hamartomatous lesions-that consist of various dental tissues such as enamel, dentin, cementum and pulp arranged in an unstructured, disorganized fashion [1]. First described in the 19th century, odontomas were initially regarded as true neoplasms but were later reclassified as hamartomas due to their limited growth potential and benign behaviour [2]. These lesions are generally asymptomatic and are most often discovered incidentally on radiographs taken for delayed eruption of permanent teeth or failure of exfoliation of deciduous teeth. In children and adolescents, the presence of complex odontomas can significantly disrupt the natural course of dental development, impeding the eruption of underlying permanent teeth and potentially leading to malocclusion or space loss [3, 4]. Radio graphically, complex odontomas appear as irregular, radiopaque masses with a radiolucent halo, typically found in the posterior jaws, more commonly in the mandible. While surgical enucleation remains the treatment of choice to eliminate the obstructive lesion, it is increasingly recognized that the treatment does not end with surgery [5]. The removal of the lesion alone often does not guarantee spontaneous eruption or proper alignment of the impacted teeth. Hence, orthodontic intervention becomes crucial in repositioning the teeth and restoring normal occlusion [6, 7, 8]. Spontaneous eruption following odontoma removal remains unpredictable, with Ashkenazi *et al.* [9] reporting a 32% eruption rate, while Tomizawa *et al.* [10] observed 45% in similar cases. Einy *et al.* [11] emphasized the importance of timely intervention, noting that 42% of impacted teeth fail to erupt after lesion removal alone. Adjunctive orthodontic traction often enhances outcomes, particularly when early surgical removal is performed [8]. Muczkowska *et al.* [12] further highlighted that younger patients show improved due to greater bone plasticity and eruptive potential. Therefore, it is of interest to assess the therapeutic benefits of a combined surgical-orthodontic approach in growing patients diagnosed with complex odontomas, with a focus on tooth eruption success, alignment and overall treatment outcomes.

## Materials and Methods:

This prospective interventional study was conducted between January 2022 and June 2023. A total of 20 growing patients (11 males and 9 females), aged between 8 and 16 years, were enrolled, all of whom were diagnosed with complex odontomas. The study aimed to evaluate the clinical effectiveness of a combined surgical and orthodontic approach in managing these lesions and facilitating the eruption and alignment of impacted permanent teeth. Inclusion criteria comprised patients with radiographic and histopathologic confirmation of complex odontoma, presence of unerupted or impacted permanent teeth attributed to the lesion and no systemic illness or contraindication for surgery. Patients were excluded if they had compound odontomas, had completed growth, or had previously undergone unsuccessful surgical or orthodontic interventions. Diagnosis was established through panoramic radiographs, with cone-beam computed tomography (CBCT) employed in selected cases to determine the precise location, size and extent of the lesion. Surgical enucleation of the odontoma was performed under local or general anesthesia depending on the complexity and patient compliance. Postoperative care involved routine follow-up visits to monitor healing and ensure the absence of complications. Orthodontic management commenced with a three-month period of passive monitoring to allow for spontaneous eruption of the previously impacted tooth. In cases where eruption was not observed within this period, guided orthodontic traction was initiated using fixed appliance therapy to facilitate proper positioning of the tooth into the dental arch. The outcomes of the intervention were evaluated based on several clinical parameters, including the time taken for the impacted tooth to erupt, the duration required for complete orthodontic alignment, the presence or absence of any complications such as infection or relapse and the final esthetic and functional results. Data were analyzed using descriptive statistical tools, with success rates expressed in percentages and time-dependent outcomes reported as mean values with standard deviation (SD). This structured methodology allowed for a comprehensive assessment of the treatment protocol's efficacy in young patients requiring multidisciplinary care for complex odontomas.

## Results:

The study included 20 growing patients, with a mean age of 12.3 ± 2.4 years, comprising 11 males and 9 females. The majority of complex odontomas were located in the maxilla (n = 12), while 8 cases were reported in the mandible. Successful eruption of impacted permanent teeth was observed in 18 out of 20 cases (90%) following surgical enucleation. The average time to eruption post-surgery was 7.6 ± 2.1 months, with slightly earlier eruption noted in younger patients (below 12 years) compared to older participants, though not statistically significant. Orthodontic alignment success was achieved in 85% (17/20 cases), with males showing a marginally better response to alignment possibly due to earlier detection and intervention. One patient experienced a mild postoperative infection, which was managed conservatively and two cases showed delayed eruption, necessitating extended orthodontic traction. Importantly, no recurrence of the odontoma was observed during the 12-month follow-up period (Table 1). In the present study, the mean age of patients was 12.3 ± 2.4 years, with a slightly higher eruption and alignment success observed in younger patients (<13 years). Gender-wise analysis revealed no significant differences in eruption or alignment success, although male participants demonstrated a marginally higher rate of successful orthodontic alignment. Regarding lesion location, 12 cases were located in the maxilla and 8 in the mandible. Eruption success was notably higher in maxillary lesions, accompanied by a faster mean eruption time (7.6 ± 2.1 months). The extent of the lesion also played a critical role. Localized or smaller complex odontomas were associated with spontaneous or timely eruption and required less orthodontic intervention. In contrast, larger or multi-tooth-interfering lesions often required guided traction and showed delayed eruption or minor complications such as infection or prolonged treatment. Lastly, time to intervention was a significant predictor of treatment outcome. Early diagnosis and prompt surgical management were positively correlated with eruption success, reduced complication rates and better esthetics and functional orthodontic results, emphasizing the importance of timely multidisciplinary care (Table 2).

## Discussion:

Complex odontomas, though benign, can significantly impact the normal eruption pattern of permanent teeth, especially in growing patients. Their asymptomatic nature often leads to delayed diagnosis, usually during routine radiographic evaluations for unerupted teeth. Timely surgical removal, followed by appropriate orthodontic management, plays a crucial role in ensuring optimal functional and esthetic outcomes [7, 8]. This study aims to assess the effectiveness of this combined approach, providing insight into treatment success rates and factors influencing prognosis in pediatric patients. The findings of the present study affirm the efficacy of a combined surgical and orthodontic approach for managing complex odontomas in growing patients. The eruption success rate of 90% and orthodontic alignment success of 85% was observed. However, a study by Ashkenazi *et al.* (2007) found that spontaneous eruption occurred in 83% of impacted teeth associated with normal-sized superlative supernumeraries and in 32% of cases involving odontomas after surgical removal 9]. Additionally, Tomizawa *et al.* reported that 47.6% of odontomas caused tooth impaction, with spontaneous eruption occurring in 45% of cases after odontoma removal [10]. These findings suggest that while spontaneous eruption is possible, adjunctive orthodontic traction may be necessary to achieve optimal alignment. Similarly, Ashkenazi *et al.* highlighted that early surgical intervention enhances the prognosis by preserving eruptive potential and reducing the risk of dental impaction [9]. Einy *et al.* discussed conservative treatment approaches for impacted teeth following surgical obstruction removal; highlighting the importance of timely intervention [11]. Approximately 42% of impacted teeth fail to erupt on their own after the removal of an obstructive lesion. Furthermore, when the impaction is associated with complex odontomas, the likelihood of spontaneous eruption following surgical excision is notably lower than that seen with the removal of supernumerary teeth [9]. Age-related outcomes in our study show that patients younger than 13 years had slightly better eruption and alignment outcomes. This aligns with the observations of Muczkowska *et al.* who emphasized that younger bone is more pliable, with higher remodelling capacity and eruptive potential, thereby facilitating spontaneous eruption and favorable orthodontic movement [12]. Additionally, research indicates that the eruption mechanism of odontomas differs from that of normal teeth, primarily due to the absence of a periodontal ligament in odontomas. The increasing size of the odontoma may lead to sequestration of the overlying bone, resulting in its eruption. This process is influenced by bone remodelling, which is more active in younger individuals, thereby facilitating the eruption of odontomas in this age group [13]. Our results also mirror Fleming *et al.* stated that younger patients respond better to conservative orthodontic forces post-excision of odontogenic lesions [14].

While gender did not show significant differences in most parameters, a marginally better orthodontic outcome was noted in males. Research indicates that males often exhibit larger craniofacial dimensions, including increased mandibular and maxillary widths, which may provide more space for tooth eruption and alignment. For instance, a study published in literature found that males had significantly larger intercanine and intermolar widths compared to females [15]. Similarly, research in the Journal of Oral and Maxillofacial Pathology reported greater mandibular intercanine distances in males [16]. These anatomical differences might contribute to the slightly improved orthodontic outcomes observed in male patients, although the clinical significance is often considered negligible. Maxillary odontomas often exhibit higher eruption success rates and faster eruption times compared to mandibular odontomas. This can be attributed to the thinner cortical bone and more favorable eruptive vectors in the maxilla, which facilitate easier spontaneous or assisted eruption. Conversely, mandibular lesions may present greater challenges due to denser bone and the proximity of neurovascular bundles, potentially complicating surgical access and orthodontic movement. Studies have shown that compound odontomas are frequently located in the anterior maxilla, while complex odontomas are more commonly found in the posterior mandible. This distribution may further influence the ease of eruption and the complexity of treatment in different jaw regions [17]. The extent of an odontoma significantly influences the complexity of treatment and the approach required for successful management. Smaller, localized odontomas typically exert minimal mechanical obstruction, facilitating quicker and more straightforward eruption of adjacent teeth. Conversely, larger odontomas that impinge on multiple tooth buds often necessitate more complex interventions, including prolonged orthodontic traction or re-intervention. This is particularly evident in cases involving extensive lesions in the posterior mandible, where the dense bone structure and proximity to vital structures can further complicate surgical access and orthodontic movement. Studies have documented that giant complex odontomas in the posterior mandible can lead to significant jaw expansion and facial asymmetry, underscoring the challenges associated with managing larger lesions in this region [18]. Our study underscores the importance of early diagnosis and timely surgical intervention in managing odontomas. Patients who underwent prompt surgical excision following radiographic detection experienced reduced eruption delays and improved alignment outcomes. This observation aligns with existing literature emphasizing that delayed intervention can compromise root development, potentially leading to ankylosis or unfavourable orthodontic results. For instance, a case report highlighted that early detection and surgical removal of odontomas are crucial to prevent complications such as delayed tooth eruption and ensure favorable outcomes. Similarly, another study reported that timely surgical removal of an odontoma in a pediatric patient facilitated the eruption of the impacted tooth, highlighting the significance of early intervention. These findings collectively advocate for the prompt identification and management of odontomas optimizing dental development and orthodontic outcomes [19]. The lack of recurrence at 12-month follow-up in our study reinforces the benign, non-invasive behavior of complex odontomas when properly enucleated, as supported by WHO classification of Head and Neck Tumors (2022) [20]. The current study demonstrates the benefits of early detection and interdisciplinary management of complex odontomas. Surgical removal alone may not suffice, particularly when the eruption path of the tooth is compromised. Orthodontic intervention ensures proper alignment and function. The eruption timeline depends on age, lesion size and tooth position. Early intervention, as done in this study, minimized the need for extraction or prosthetic replacement. The primary limitation of this study was the relatively small sample size and short follow-up duration of 12 months, which may not capture late recurrences or long-term alignment stability. Additionally, variations in individual healing and growth potential were not quantitatively assessed. Future multi center studies with larger cohorts, extended follow-up and inclusion of three-dimensional imaging and bone density analysis could offer deeper insights into eruption dynamics and treatment optimization.

## Conclusion:

The combined surgical and orthodontic approach proves highly effective in managing complex odontomas in growing patients, particularly when initiated early. Favorable outcomes are more frequently associated with maxillary lesions, younger age, and localized presentations. Timely yet multidisciplinary intervention is crucial for optimal eruption, alignment, and long-term functional and esthetic results.

## Figures and Tables

**Table 1:** Clinical parameters and observations

**Parameter**	**Observation**
Mean Age	12.3 ± 2.4 years
Gender Distribution	11 males, 9 females
Lesion Location	Maxilla: 12 cases, Mandible: 8 cases
Eruption Success Rate	90% (18 out of 20 cases)
Mean Eruption Time Post-Surgery	7.6 ± 2.1 months
Orthodontic Alignment Success	85% (17 out of 20 cases)
Complications Observed	1 mild infection, 2 delayed eruption cases
Recurrence at 12-Month Follow-Up	0%

**Table 2:** Correlation between clinical variables and outcomes

**Parameter**	**Eruption Success**	**Mean Eruption Time**	**Orthodontic Alignment Success**
Age	Higher success in <13 years	Slightly faster in younger group	Better alignment in younger patients
Gender	No significant difference	Similar across genders	Males showed marginally better results
Lesion Location	Maxilla > Mandible	Maxilla: Faster eruption	Maxillary cases easier to align
Extent of Lesion	Localized lesions: Higher success	Smaller lesions: Faster eruption	Extensive lesions needed longer treatment
Time to Intervention	Early treatment better success	Early surgery reduced delay	Early intervention better alignment

## References

[1] Tyagi P, Singla S (2010). Int J Clin Pediatr Dent..

[2] Ide F (2023). Head Neck Pathol..

[3] Kulkarni V.K (2013). BMJ Case Rep..

[4] Das U.M (2009). Int J Clin Pediatr Dent..

[5] Alarcön Apablaza J (2024). Medicina (Kaunas)..

[6] Mahardawi B (2020). J Korean Assoc. Oral Maxillofac Surg..

[7] da Silva V.A (2019). J Clin Exp Dent..

[8] Althobaiti F.H (2025). Cureus..

[9] Ashkenazi M (2007). Am J Orthod Dentofacial Orthop..

[10] Tomizawa M (2005). Int J Paediatr Dent..

[11] Einy S (2022). Applied Sciences..

[12] Muczkowska N (2025). Dentistry Journal..

[13] Ahmed K.A (2015). Saudi Med J..

[14] Fleming P.S (2015). Cochrane Database Syst Rev..

[15] Lubis H.F (2022). Journal of Orthodontic Science..

[16] Krishnan R.P (2024). Journal of Oral and Maxillofacial Pathology..

[17] DeColibus K.A (2023). Dentistry Journal..

[18] Park J.C (2018). Imaging Sci Dent..

[19] Bawazir O.A (2021). Journal of Contemporary Dental Practice..

[20] https://publications.iarc.who.int/Book-And-Report-Series/.

